# Factors that predict weight loss success differ by diet intervention type

**DOI:** 10.3389/fnut.2023.1192747

**Published:** 2023-07-17

**Authors:** Jordan Losavio, Michael J. Keenan, Elizabeth A. Gollub, Heidi J. Silver

**Affiliations:** ^1^College of Agriculture, Louisiana State University, Baton Rouge, LA, United States; ^2^Louisiana State University Agricultural Center, Baton Rouge, LA, United States; ^3^Department of Medicine, Vanderbilt University Medical Center, Nashville, TN, United States; ^4^Department of Veterans Affairs, Tennessee Valley Healthcare System, Nashville, TN, United States

**Keywords:** obesity, diet, fat, carbohydrate, weight loss, cardiometabolic

## Abstract

**Background:**

Many types of diet intervention can achieve negative energy balance and successful weight loss in persons with obesity. However, within any dietary strategy, there is large inter-individual variation in the weight loss response. The aim of this study is to determine factors that predict weight loss success for diet interventions that vary by macronutrient and caloric composition.

**Methods:**

Participants with BMI 30.0 to 49.9 kg/m^2^ self-selected one of three diet intervention trials for weight loss: low carbohydrate (LOW CHO), low fat (LOW FAT), or low calorie (LOW KCAL). Multivariable regression models were developed to determine the significance of predictor demographic, body composition, metabolic, clinical, and dietary variables for each diet type.

**Results:**

Weight loss over 12–16 weeks averaging −5.1 ± 4.0 kg from baseline weight, *p* < 0.001, was not significantly different among diet types. Several different factors were identified that account for the inter-individual variance in weight loss success. Regardless of diet type, the most robust predictor of weight loss success was completion of the intervention, accounting for 20–30% of the variance. Factors predicting diet intervention completion were age, physical activity level, blood leptin level, blood pressure, and the amount of weight loss occurring. Differences by diet type in cardiometabolic risk factor reduction were identified with LOW CHO decreasing glycemia/insulinemia factors, LOW FAT decreasing lipidemia factors, and LOW KCAL decreasing inflammatory factors.

**Conclusion:**

These data provide evidence to inform more precise and personalized approaches to diet intervention for weight loss and cardiometabolic health.

## 1. Introduction

Overweight and obesity is affecting ~2 billion adults and almost 400 million children and adolescents globally (1). A market research survey of 22,008 individuals from 30 countries showed that 45–60% of people are currently trying to lose weight (2). These statistics underscore the urgent need to identify key determinants for efficacy of weight loss diets (3). While many types of diet interventions can achieve a negative energy balance, a successful dietary strategy is one that facilitates a loss of at least 5% of baseline body weight and improves cardiometabolic health (4). Common dietary strategies include varying macronutrient composition (e.g., low carbohydrate or low fat) as well as restricting total energy (low calorie) intake. Low carbohydrate diets are often promoted for weight loss based on the hypothesis that they increase satiety and promote lipolysis, as illustrated by the carbohydrate insulin model (5). Low fat diets have been recommended as a means for avoiding positive energy balance and reducing cardiovascular disease risk (6). Low calorie diets promote negative energy balance, but may be thwarted by adaptative thermogenesis that favors weight regain (7).

A meta-analysis of 5 randomized controlled trials that included 447 free-living adults showed that weight loss from low carbohydrate diets was greater than from low fat diets by 3.3 kg at the 6-month timepoint, but this difference was not maintained at 12 months (8). More recently, a meta-analysis of 121 randomized controlled trials that included 21,942 overweight/obese adults, similar in age to those in the prior meta-analysis, was conducted (9). This analysis demonstrated minimal differences in weight loss at 6 months between low carbohydrate and low fat diet types, and no significant differences at 12 months (9). These findings indicate that weight loss can be achieved with adherence to any diet type (10).

However, within any dietary strategy, there is large variation in weight loss response among individuals. For example, in a 12-month randomized trial comparing the efficacy of four popular diets, weight change among participants within a given diet type ranged from −30 to +10 kilograms (11). This inter-individual variability may be accounted for by a range of biological, physiological, psychological, behavioral, and environmental factors. The purpose of the present study is to determine factors that impact weight loss and weight loss success in diet interventions that vary by macronutrient and caloric content. These findings may assist in personalizing dietary approaches for weight loss and optimizing treatment outcomes.

## 2. Methods

### 2.1. Recruitment and eligibility

Participants were recruited for dietary weight loss interventions that were conducted at Vanderbilt University Medical Center (VUMC) between 2016–2020 (Supplementary Figure S1) upon responding to flyers posted in the metropolitan Nashville area at college campuses, public libraries, community parks, and community agency offices or responding to a study-specific announcement distributed on the VUMC research email listserv. To be included participants were age 21–60 years, BMI between 30.0 to 49.9 kg/m^2^, and weight stable during the 3 months prior to enrollment. Exclusion criteria were diagnosis in the electronic medical record of esophageal disorders, type 1 and 2 diabetes, cancer, liver disease, respiratory disease, kidney disease, cardiovascular disease, uncontrolled hypertension, or a history of esophageal or bariatric surgery. Potential participants were also excluded if they had food allergies or dietary restrictions, gastrointestinal malabsorption, alcohol consumption averaging >2 drinks/day during the 3 months prior to enrollment, had a history of smoking, vaping, or illicit drug use, taking medications that alter appetite or energy metabolism, or were pregnant or lactating. The studies were approved by the Vanderbilt University Medical Center Institutional Review Board and all participants signed written informed consent.

### 2.2. Diet interventions

**Figure 1:**
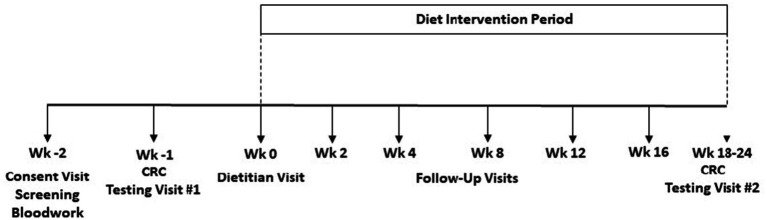
Study design.

To mimic real-world conditions, participants self-selected one of three diet intervention types (Supplementary Figure S1). The diet interventions were 4–6 months in duration (Figure 1) and utilized a dietary strategy with defined macronutrient distributions and/or energy restriction. A 7-day rotation of menus was developed for each diet type by research dietitians at the Vanderbilt Diet, Body Composition and Human Metabolism Core (Core) using Nutrition Data System for Research software (NDS-R version 2015, Nutrition Coordinating Center, Minn., MN) to assure that each diet type met the planned macronutrient and energy goals. To establish individual caloric goals for weight loss, resting energy expenditure was measured by metabolic cart (ParvoMedics TrueOne 2,400^®^, Sandy, UT) and multiplied by an activity factor determined by a subject’s total physical activity score. The low carbohydrate (LOW CHO) diet menus were designed to provide 30% of energy from carbohydrate, 50% of energy from fat, and 20% of energy from protein. The low fat (LOW FAT) and low calorie (LOW KCAL) diet menus were designed to provide 50% of energy from carbohydrate, 30% of energy from fat, and 20% of energy from protein. In addition, the LOW KCAL diet was designed to reduce baseline habitual energy intake by 500 calories per day. During the consent visit, participants agreed to refrain from heavy alcohol consumption and vigorous physical activity during the diet intervention period.

### 2.3. Diet assessment

Dietary intakes were assessed for energy and nutrient composition by 24-h diet recall interviews conducted by registered dietitians at the Core using the validated U.S. Department of Agriculture five-step multi-pass methodology, a standardized questionnaire, and computer-generated prompts from NDS-R version 2016 (12, 13). Direct entry into NDS-R enabled identifying foods and beverages consumed by name, brand, and preparation method from a database of 18,000 items. Portion sizes of all foods and beverages consumed during the 24-h periods were estimated using standard measuring utensils (plates, cups, bowls, and spoons of various sizes). Data were analyzed for energy (kcal), energy density (kcal/g), macronutrients as percentage of energy, and two micronutrients (sodium and potassium) due to their role in hypertension, the major risk factor for cardiovascular disease (14). Food level data were also categorized into 12 subgroups: juices and sugar sweetened beverages, fruit and non-starchy vegetables, starchy vegetables, fats and fried foods, plant proteins, animal proteins, dairy, snacks and desserts, whole grains, refined grains, artificially sweetened beverages, and alcohol for analysis (Supplementary Table S1).

### 2.4. Anthropometry, body composition, and resting energy expenditure

Participants were instructed to avoid alcohol, excess caffeine intake, and non-routine physical activities on the day before each testing visit at the Vanderbilt Clinical Research Center, and to fast from 9:00 pm until arrival at 7:00 am. After vital signs were obtained, height (± 0.1 cm), weight (± 0.1 kg), and waist and hip circumferences (± 0.1 cm) were measured in triplicate using standardized procedures. Participants then rested in the supine position for ≥10 min prior to data collection for resting energy expenditure (REE) and substrate oxidation rates. We used a metabolic cart system (ParvoMedics TrueOne 2,400®, Sandy, UT) that was calibrated to room air and a single gas tank prior to data collection. Data was collected for 15 min at steady state under the hood with average change in minute VO_2_ ≤ 10% and respiratory quotient (RQ) ≤ 5%. REE was calculated via the Weir equation and substrate oxidation was determined according to the method of Frayn upon adjustment for 24-h urinary urea nitrogen output (15, 16).

Dual energy x-ray absorptiometry (DXA) was used to measure body composition using a Lunar iDXA scanner (GE Healthcare) with Encore software (version 13.6). Measurements were performed by one trained certified densitometrist after machine calibration to a phantom. Outputs included visceral adipose tissue (VAT) mass, total and regional fat mass, total and regional lean mass, and bone mineral area and density. In comparing performance of our DXA protocol *vs* whole body MRI, we show coefficients of variation <1.5% for total fat, trunk fat, total lean, and trunk lean masses, indicating good precision and reliability of the DXA data (17).

### 2.5. Clinical biomarkers

Whole blood and 24-h urine samples were collected at the clinical research center. A urine β-hCG test confirmed non-pregnancy at each study visit. Plasma glucose (colorimetric timed endpoint method), serum insulin (chemiluminescent immunoassay), plasma lipid profiles (selective enzymatic hydrolysis), and plasma C-reactive protein (CRP via enhanced turbidimetric assay) levels were measured at the Vanderbilt Department of Pathology Diagnostic Laboratory. Plasma leptin levels were measured at the Vanderbilt Hormone Core by radioimmunoassay. HOMA-IR was calculated from measured glucose and insulin levels [(fasting glucose (mg/dL) × fasting insulin (mU/mL)) / 405]. Metabolic syndrome status was determined based on having abnormal levels for ≥3 of the 5 established risk criteria (18).

### 2.6. Questionnaires

The three subscales of the Eating Attitudes Test (EAT-26) were scored with 20 or higher indicating high eating disorder risk characterized by high level of preoccupation with body weight, body image, and eating (19). Depression was categorized as “none to mild” versus “moderate to severe” based on scoring ≥16 on the Center for Epidemiological Studies Depression (CES-D) scale or ≥ 10 on the Beck Depression Inventory (20). Physical activity levels were established from scores on the Physical Activity during Cancer Treatment Questionnaire (PACT-Q) and the Baecke Physical Activity Questionnaire (21).

### 2.7. Statistical analysis

Differences in baseline characteristics among the 3 diet types were tested by chi-square or ANOVA. As a result of this testing sex, age, and BMI were included as covariates in all modeling. Potential predictors for each diet type were identified by univariate linear regression analysis with amount of weight loss as the primary outcome. Multivariable linear regression models were developed to determine the significance of predictor variables for each diet type. Prior to analysis, mean imputation was utilized to replace missing values, which comprised <1% of the independent variables (22). Akaike information criterion (AIC) forward-backward stepwise regression was used to remove nonsignificant covariates while achieving the best fit of the data. Initially, food and nutrient variables were excluded, leaving 26 independent variables. Dietary variables that were significant in the univariate analysis were then added to the multivariate model if this improved model performance (*R*^2^ increased by ≥1%). The Variance Inflation Factor (VIF) was capped at 5 to reduce potential multicollinearity. The percentage of the variance explained by each predictor variable was estimated by dividing the individual sum of squares by the total sum of squares for all variables and residuals. This value was used to determine relative influence of each predictive factor on amount of weight loss. Logistic regression models for each diet type were also created for the secondary outcomes of: (a) weight loss success (yes/no), defined as weight loss ≥5% of baseline body weight and (b) diet intervention completer (yes/no). Lastly, we performed exploratory analysis to determine any trends within each diet type for improvements in clinical biomarkers of cardiometabolic health via analysis of covariance (ANCOVA) with the baseline biomarker value as a covariate. RStudio for Windows software (R Foundation for Statistical Computing, Vienna, Austria) was used for all statistical analyses with a type I error of 5% (23).

## 3. Results

Of the 305 study participants who met enrollment criteria (Supplementary Figure S1), 65% self-identified as white and 35% as Black or other, with no significant differences among diet type groups by race/ethnicity, income, marital status, or educational status (Table 1). Unlike the LOW FAT and LOW KCAL diet groups, the LOW CHO group, comprising 47% of all participants, was 100% female. At baseline, the LOW KCAL group was older, had higher glucose and insulin levels, and had a greater proportion of participants meeting metabolic syndrome criteria compared to the LOW CHO and LOW FAT diet groups (all *p*s < 0.001). Overall, 48% of participants met BMI criteria for Class I obesity (BMI 30.0–34.9 kg/m^2^), 42% for Class II obesity (BMI 35.0–39.9 kg/m^2^), and 10% for Class III obesity (BMI ≥ 40.0 kg/m^2^).

**Table 1:** Baseline descriptive characteristics of study participants by diet type.

	LOW CHO	LOW FAT	LOW KCAL	ALL	
	*N* = 144	*N* = 85	*N* = 76	*N* = 305	*p*-value
*Demographics*					
Age (year)	36.8 ± 6.8^a^	38.7 ± 8.2^a^	48.0 ± 7.0^b^	39.9 ± 8.5	< 0.001
Sex (male)	0 (0%)^a^	21 (25%)^b^	23 (35%)^b^	44 (15%)	< 0.001
Race (non-white)	41 (29%)	34 (39%)	29 (44%)	104 (35%)	0.06
Education					0.24
High School or less	22 (15%)	9 (11%)	11 (17%)	42 (14%)	
College or more	120 (85%)	76 (89%)	55 (83%)	251(86%)	
Income					0.65
≤ $50 k/yr.	67 (47%)	38 (45%)	33 (50%)	138 (47%)	
> $50 k/yr.	75 (53%)	47 (55%)	33 (50%)	155 (53%)	
Married (yes)	83 (58%)	47 (55%)	44 (67%)	174 (59%)	0.26
*Anthropometrics*					
Height (cm)	163.5 ± 6.4^a^	167.4 ± 8.2^b^	169.4 ± 9.4^b^	166.0 ± 8.1	< 0.001
Weight (kg)	93.0 ± 10.6^a^	99.7 ± 13.5^b^	107.1 ± 17.0^c^	98.1 ± 14.2	< 0.001
Body Mass Index (kg/m^2^)	34.8 ± 2.7^a^	35.6 ± 3.3^a^	36.9 ± 4.2^b^	35.5 ± 3.3	< 0.001
Body Fat (%)	47.2 ± 3.4^a^	45.5 ± 6.4^b^	45.1 ± 5.6^b^	46.2 ± 5.0	0.006
*Clinical Biomarkers*					
Glucose (mg/dL)	92.3 ± 12.1^a^	89.6 ± 8.5^a^	144.9 ± 43.0^b^	103.4 ± 30.5	< 0.001
Insulin (mIU/L)	10.7 ± 7.5^a^	10.2 ± 5.9^a^	29.4 ± 14.5^b^	14.8 ± 11.8	< 0.001
HOMA-IR (score)	2.6 ± 2.1^a^	2.3 ± 1.4^a^	10.3 ± 5.3^b^	4.2 ± 4.3	< 0.001
TG/HDL-Cholesterol (ratio)	2.1 ± 1.4^a^	2.1 ± 1.3^a^	3.7 ± 2.3^b^	2.4 ± 1.8	< 0.001
LDL–Cholesterol (mg/dL)	102.3 ± 25.4	106.2 ± 28.1	105.3 ± 33.1	104.1 ± 28.0	0.56
C-Reactive Protein (mg/L)	6.0 ± 6.9	4.8 ± 4.7	6.7 ± 7.1	5.8 ± 6.3	0.18
Systolic Pressure (mmHg)	121.4 ± 11.1	124.6 ± 13	123.1 ± 13.9	122.7 ± 12.1	0.15
Diastolic Pressure (mmHg)	69.9 ± 8.5^a^	74.3 ± 9.5^b^	69.6 ± 9.1^a^	71.1 ± 8.9	< 0.001
Resting Energy Expenditure (kcal)	1608.2 ± 191.9^a^	1638.8 ± 314.1^a^	1890.5 ± 303.4^b^	1680.7 ± 277.3	< 0.001
Respiratory Quotient (VCO_2_/VO_2_)	0.82 ± 0.05	0.82 ± 0.09	0.80 ± 0.05	0.82 ± 0.06	0.16
Leptin (ng/mL)	33.8 ± 9.8^a^	22.8 ± 9.9^b^	30.1 ± 17.1^a^	29.8 ± 12.0	< 0.001
*Risk factors*					
Metabolic Syndrome (yes)	40 (28%)^a^	22 (26%)^a^	58 (76%)^b^	117 (38%)	< 0.001
Low Physical Activity (yes)^*^	36 (25%)	21 (25%)	19 (26%)	76 (25%)	0.94
Mod-Severe Depression (yes)^**^	11 (8%)	6 (7%)	4 (6%)	21 (7%)	0.91
Eating Behavior (score)^***^	12.5 ± 7.6^a^	10.0 ± 6.0^b^	10.3 ± 5.1^b^	11.3 ± 6.8	0.009

^*^Low physical activity is defined as being in the bottom quartile of physical activity scores (21). ^**^Moderate to severe depression is determined by score on the CESD/BDI scales (20). ^***^Eating behavior score is based on the EAT-26 questionnaire (19). ^****^Values with different superscript letters (^a b c^) are significantly different.

At baseline, prior to any diet intervention, reported intakes of the amount of energy (kcal) consumed differed among the groups (*p* < 0.001), ranging from 1903.0 ± 447.5 kcal/day in the LOW CHO group to 2106.1 ± 970.7 kcal/day in the LOW FAT group to 2349.9 ± 707.4 kcal/day in the LOW KCAL group (Table 2). However, there were no significant differences observed among diet groups for the macronutrient composition (percentage of energy as fat, carbohydrate, and protein) of habitual dietary intakes. The LOW CHO group reported consuming fewer simple carbohydrates (simple sugars), fewer servings/day of refined grains, fewer servings/day of juice and sugar-sweetened beverages, fewer servings/day of snacks and desserts, and less sodium. The LOW FAT group reported a higher intake of monounsaturated fats and fewer servings/day of total fats, fried foods, and fast foods. The LOW KCAL group reported a higher intake of dietary fiber, more servings/day of refined grains, a higher glycemic load, and higher sodium intake.

**Table 2:** Reported dietary intakes at baseline by diet type.^*^

	LOW CHO	LOW FAT	LOW KCAL	
	*N* = 144	*N* = 85	*N* = 76	*p*-value
*Nutrients*				
Total Energy (kcal)	1903.0 ± 447.5^a^	2106.1 ± 970.7^a,b^	2349.9 ± 707.4^b^	< 0.001
Energy Density (kcal/g)	0.7 ± 0.2^a^	0.9 ± 0.4^b^	0.7 ± 0.3^a^	0.01
Fat (%)	38.4 ± 7.4	38.0 ± 11.9	40.8 ± 9.4	0.14
Carbohydrate (%)	44.5 ± 8.8	46.4 ± 14.0	42.5 ± 10.4	0.09
Protein (%)	16.6 ± 4.3	16.1 ± 5.3	16.0 ± 4.3	0.54
Animal Protein (%)	67.1 ± 12.0	66.2 ± 16.5	65.3 ± 26.0	0.69
Saturated Fat (g)	28.0 ± 9.7	30.6 ± 21.5	33.1 ± 11.9	0.05
Polyunsaturated Fat (g)	18.1 ± 8.9^a^	19.1 ± 13.6^a^	37.7 ± 14.5^b^	< 0.001
Monounsaturated Fat (g)	30.2 ± 9.3^a,b^	33.1 ± 19.4^a^	26.5 ± 15.1^b^	0.02
Omega-3 Fatty Acids (g)	1.8 ± 0.9^a^	2.0 ± 1.3^a^	2.5 ± 1.5^b^	< 0.001
Simple Sugars (g)	87.2 ± 44.3^a^	112.4 ± 100.8^b^	97.7 ± 69.1^a,b^	0.03
Starch (g)	103.0 ± 36.8	109.1 ± 60.7	117.8 ± 52.9	0.12
Fiber (g)	15.6 ± 6.0^a^	15.1 ± 8.4^a^	19.4 ± 10.1^b^	0.001
Glycemic Load	121.3 ± 41^a^	142.1 ± 84.7^b^	146.6 ± 69.1^b^	0.01
Sodium (mg)	3412.9 ± 982.3^a^	3450.2 ± 1668.8^a^	4431.9 ± 1711.6^b^	< 0.001
Sodium:Potassium Ratio	1.7 ± 0.7	1.6 ± 0.7	1.8 ± 0.7	0.18
*Foods (svgs/day)*				
Fruits & Non-Starchy Vegetables	3.2 ± 1.5	2.5 ± 2.4	2.9 ± 3.2	0.15
Starchy Vegetables	0.3 ± 0.3	0.2 ± 0.5	0.4 ± 0.7	0.23
Fats, Fried Foods, Fast Foods	6.9 ± 5.1^a^	4.8 ± 4.2^b^	5.7 ± 4.3^a,b^	0.003
Plant Proteins	1.6 ± 1.0^a^	0.7 ± 1.6^b^	0.9 ± 2.2^b^	< 0.001
Animal Proteins	5.2 ± 2.1	5.0 ± 3.2	5.7 ± 3.8	0.36
Dairy Products	1.3 ± 0.6	1.4 ± 2.0	1.3 ± 1.1	0.72
Whole Grains	0.9 ± 0.9	1.1 ± 1.7	0.9 ± 1.3	0.72
Refined Grains	3.4 ± 2.1^a^	4.6 ± 3.2^b^	5.0 ± 3.1^b^	< 0.001
Snacks & Dessert items	0.9 ± 1.1^a^	2.2 ± 3.0^b^	1.6 ± 1.6^b^	< 0.001
Juice & Sugar-Sweetened Beverages	0.6 ± 1.0^a^	1.3 ± 1.7^b^	1.0 ± 1.8^b^	< 0.001
Artificially Sweetened Beverages	0.8 ± 1.5	0.8 ± 1.6	1.1 ± 2.1	0.32
Alcohol Beverages	0.1 ± 0.4	0.1 ± 0.5	0.0 ± 0.1	0.10

Values with different superscript letters ^(a b)^ are significantly different.

### 3.1. Impact of intervention on body weight by diet type

**Figure 2:**
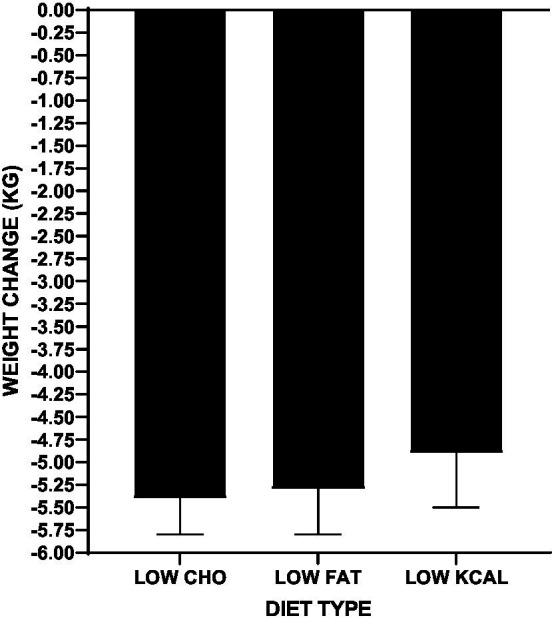
Comparison among diet types for total weight change.

Participants in all diet types experienced a significant weight loss averaging 5.1 ± 4.0 kg from baseline weight (*p* < 0.001). Neither the amount or rate of weight loss were significantly differently among the diet types: LOW CHO -5.0 ± 4.0 kg; LOW FAT -5.2 ± 3.9 kg; LOW KCAL -4.9 ± 4.2 kg, *p* = 0.85 (Figure 2; Supplementary Figure S2). There was also no significant difference in the proportion of participants who achieved successful weight loss (≥ 5% of baseline weight), with 49% of the LOW CHO group, 51% of the LOW FAT group, and 38% of the LOW KCAL group achieving weight loss success (*p* = 0.23). Yet, participants who completed all weeks of their respective diet intervention type lost more weight than non-completers (LOW CHO -6.7 ± 4.0 *vs* − 2.1 ± 1.9 kg; LOW FAT -6.1 ± 3.5 *vs* − 1.4 ± 2.97 kg; LOW KCAL -6.0 ± 4.2 *vs* − 1.7 ± 2.2 kg, all *p*s < 0.001). Univariate analysis showed that amount of weight loss, older age, higher leptin level, higher physical activity score, and lower depression score were associated with diet intervention completion (Supplementary Table S1). Although diet type was not significantly associated with completion status, there was a difference by diet type in the proportion of participants who completed all study weeks (LOW CHO 64%, LOW FAT 81%, LOW KCAL 74%, *p* = 0.02).

### 3.2. Predictors of amount of weight loss by diet type

As expected, there was much variability in weight change among participants within each diet type. Linear regression modeling was used to determine the most parsimonious models to predict amount of weight loss for each diet type. For the LOW CHO diet, the factors that accounted for 41% of the inter-individual variance in weight loss were completion status, self-reported race, baseline percent body fat, respiratory quotient, and metabolic syndrome status. For the LOW FAT diet, 51% of the inter-individual variance in weight loss was accounted for by completion status, education level, marital status, baseline percent body fat, LDL-cholesterol level, leptin level, blood pressure, resting energy expenditure, and fruit and vegetable intake. For the LOW KCAL diet, 42% of the inter-individual variability in weight loss was accounted for by completion status, age, education level, LDL-cholesterol level, insulin level, systolic blood pressure, leptin level, eating behavior score, metabolic syndrome status, and protein and simple sugars intake. Completion status accounted for the greatest proportion of the inter-individual variance in all diet types (Table 3).

**Table 3:** Results from multivariable linear regression modeling to predict amount of weight change by diet type.^*^

Predictors	% Variance	Estimate	std. error	Statistic	*p*-value
**LOW CHO diet: Adj. *R***^**2**^ **= 0.40, *p* < 0.001**					
(Intercept)		26.449	6.270	4.22	<0.001
Completion (no/yes)	29.84	4.818	0.579	8.32	<0.001
Race (white/non-white)	4.56	−1.978	0.636	−3.12	0.002
Body Fat (%)	1.01	−0.195	0.086	−2.28	0.024
Respiratory Quotient (VCO_2_/VO_2_)	3.03	−17.295	5.649	−3.06	0.003
Metabolic Syndrome (no/yes)	2.90	−1.866	0.311	−2.79	0.006
**LOW FAT diet: Adj. *R***^**2**^ **= 0.51, *p* < 0.001**					
(Intercept)		0.737	5.391	0.13	0.892
Completion (no/yes)	23.92	3.664	0.868	4.22	< 0.001
Education (high school/college)	7.28	2.108	1.036	2.04	0.045
Marital Status (no/yes)	0.04	0.982	0.664	1.48	0.143
Body Fat (%)	0.45	0.153	0.076	2.02	0.047
LDL-Cholesterol (mg/dL)	6.58	−0.040	0.012	−3.44	<0.001
Diastolic Pressure (mm Hg)	3.62	−0.130	0.048	−2.74	0.008
Systolic Pressure (mm Hg)	1.43	0.084	0.037	2.25	0.027
Resting Energy Expenditure (kcal)	1.97	−0.003	0.001	−1.99	0.051
Leptin (ng/dL)	10.34	−0.173	0.045	−3.86	< 0.001
Fruit & Vegetable Intake (svgs/day)	3.05	0.346	0.127	2.72	0.008
**LOW KCAL diet: Adj. *R***^**2**^ **= 0.42, *p* < 0.001**					
(Intercept)		−3.350	5.382	−0.62	0.536
Completion (no/yes)	19.54	3.662	0.871	4.21	<0.001
Age (years)	3.01	0.143	0.057	2.52	0.015
Education (high school/college)	3.54	−2.337	1.039	−2.25	0.029
Insulin (mIU/L)	1.04	0.038	0.029	1.31	0.197
LDL-Cholesterol (mg/dL)	3.71	0.035	0.012	2.94	0.005
Systolic Pressure (mm Hg)	2.27	−0.050	0.033	−1.54	0.129
Leptin (ng/dL)	6.59	−0.044	0.027	−1.63	0.110
Metabolic Syndrome (no/yes)	2.76	−0.768	0.442	−1.74	0.088
EAT-26 (score)	4.81	0.118	0.075	1.58	0.121
Protein Intake (% kcal)	2.12	0.170	0.100	1.69	0.096
Simple Sugars Intake (g/day)	8.70	−0.014	0.006	−1.69	0.096

^*^Multiple linear regression and variable selection were performed to generate the best fitting model for the outcome of amount of weight loss for each diet type.

### 3.3. Predictors of successful weight loss (≥ 5% baseline weight) by diet type

Logistic regression modeling was used to predict weight loss success by diet type (Table 4). For the LOW CHO diet, completion status, age, self-reported race, baseline percent body fat, and baseline glucose level accounted for 41% of the variance in weight loss success. For the LOW FAT diet, completion status, baseline insulin level, LDL-cholesterol level, physical activity score, and fruit and vegetable intake accounted for 40% of the variance in weight loss success. For the LOW KCAL diet, completion status, age, LDL-cholesterol level, and leptin level accounted for 24% of the variance in weight loss success. As with the linear regression modeling for amount of weight loss, completion of the diet intervention most significantly increased the odds for weight loss success with all three diet types [LOW CHO: OR 44.87 (13.22, 152.26); LOW FAT: OR 64.0 (6.17, 664.4); LOW KCAL: OR 17.42 (1.95, 155.71)]. The strongest predictor for diet intervention completion was the amount of weight loss being achieved [OR 1.58 (CI 1.39, 1.80)]. Other predictors of diet intervention completion were age, physical activity score, leptin level, and systolic blood pressure (Supplementary Table S3). Diet type was not a significant predictor of completion.

**Table 4:** Results from logistic regression modeling to predict achieving successful weight loss (≥ 5% of baseline weight) by diet type.^*^

Predictors	OR	95% CI	Increment	Wald statistic	*p*-value
**LOW CHO diet: *R***^**2**^ **= 0.41**					
(Intercept)				4.61	0.032
Completion	44.87	13.22, 152.26	Completer	37.23	<0.001
Age	0.93	0.87, 1.00	Year	3.94	0.047
Race (self-reported)	0.20	0.07, 0.58	Non-white	8.81	0.003
Body Fat %	0.80	0.66, 0.91	Percent	7.99	0.005
Glucose	1.03	1.00, 1.07	mg/dL	2.82	0.093
**LOW FAT diet: *R***^**2**^ **= 0.40**					
(Intercept)				1.48	0.401
Completion	64.03	6.17, 664.4	Completer	12.14	<0.001
Insulin	0.89	0.79, 0.99	mIU/L	4.15	0.042
LDL-Cholesterol	0.96	0.94, 0.99	mg/dL	10.16	0.001
Physical Activity	0.03	0.01, 1.56	Score	3.01	0.083
Fruit & Vegetable Intake	1.46	1.06, 2.01	Serving	5.22	0.022
**LOW KCAL diet: *R***^**2**^ **= 0.24**					
(Intercept)				8.05	0.005
Completion	17.42	1.95, 155.71	Completer	6.54	0.011
Age	1.13	1.02, 1.25	Year	5.18	0.023
LDL-Cholesterol	1.02	0.99, 1.04	mg/dL	2.19	0.139
Leptin	0.95	0.91, 1.00	mg/dL	3.50	0.061

^*^Logistic regression modeling and variable selection were performed to generate the best fitting model for the outcome of weight loss success (≥ 5% of baseline weight) for each diet type.

### 3.4. Impact of weight loss on cardiometabolic biomarkers by diet type

Exploratory analysis was performed to uncover trends within each diet type on nine fundamental biomarkers of cardiometabolic health (Table 5). Participants with successful weight loss in the LOW CHO group had significantly improved blood insulin level, HOMA-IR score, LDL-cholesterol level, and systolic and diastolic blood pressure. For participants with successful weight loss in the LOW FAT group, there were significantly improved LDL-cholesterol level, triglyceride level, TG/HDL ratio, and diastolic blood pressure. In the LOW KCAL group, participants with successful weight loss had significantly improved blood glucose level, HDL-cholesterol level, systolic and diastolic blood pressure, and serum CRP level.

**Table 5:** Comparison of changes in cardiometabolic biomarkers by diet type.

	Weight loss ≥5%			Weight loss <5%			p for Difference in mean change between groups
	Baseline	Final	*p*-value	Baseline	Final	*p*-value	
Low CHO Diet	*N* = 63			*N* = 26			
Glucose	93.4 ± 11.5	91.1 ± 7.5	0.07	88.2 ± 7.4	92.4 ± 8.8	0.01	0.04
Insulin	10.7 ± 7.6	8.1 ± 5.3	<0.001	8.6 ± 5.2	8.1 ± 5.1	0.49	0.16
HOMA-IR score	2.6 ± 2.1	1.9 ± 1.3	<0.001	1.8 ± 1.0	1.8 ± 1.2	0.94	0.22
LDL-Cholesterol	104.2 ± 22.5	96.4 ± 20.6	0.001	105.0 ± 31.1	99.81 ± 28.4	0.05	0.41
HDL-Cholesterol	46.6 ± 11.2	46.1 ± 10.1	0.61	50.0 ± 11.4	50.0 ± 8.4	0.89	0.53
Triglycerides	88.9 ± 47.6	81.7 ± 47.7	0.13	85.4 ± 31.9	89.7 ± 39.8	0.46	0.09
TG/HDL ratio	2.1 ± 1.5	1.9 ± 1.4	0.20	1.8 ± 0.9	1.8 ± 0.9	0.99	0.69
Systolic Pressure	120.6 ± 10.7	112.3 ± 10.4	<0.001	123.9 ± 10.8	120.4 ± 9.1	0.03	< 0.001
Diastolic Pressure	69.5 ± 8.4	64.6 ± 7.8	<0.001	71.9 ± 8.1	70.8 ± 5.2	0.38	< 0.001
C-Reactive Protein	6.2 ± 7.1	5.5 ± 5.4	0.33	4.9 ± 3.5	3.9 ± 3.3	0.10	0.33
Metabolic Syndrome Score	2.2 ± 0.9	1.9 ± 0.7	0.03	1.9 ± 0.9	2.0 ± 0.7	0.75	0.16

	Weight loss ≥5%			Weight loss <5%			p for Difference in mean change between groups
	Baseline	Final	*p*-value	Baseline	Final	*p*-value	
Low FAT Diet	*N* = 39			*N* = 24			
Glucose	89.7 ± 9.1	90.1 ± 8.2	0.81	90.4 ± 8.6	92.0 ± 11.11	0.54	0.46
Insulin	8.5 ± 5.2	7.9 ± 3.6	0.42	10.9 ± 5.6	12.6 ± 7.4	0.19	0.01
HOMA-IR score	1.9 ± 1.4	1.7 ± 0.8	0.40	2.3 ± 1.2	2.5 ± 1.0	0.45	< 0.001
LDL-Cholesterol	107.7 ± 22.8	100.7 ± 22.8	0.002	114.9 ± 28.8	113.0 ± 31.6	0.67	0.04
HDL-Cholesterol	52.4 ± 12.3	51.1 ± 10.8	0.28	50.4 ± 14.3	49.8 ± 14.0	0.70	0.41
Triglycerides	98.7 ± 40.8	85.2 ± 29.9	0.009	99.9 ± 49.0	105.3 ± 53.1	0.48	0.03
TG/HDL ratio	2.1 ± 1.4	1.8 ± 0.8	0.02	2.2 ± 1.6	2.4 ± 1.6	0.35	< 0.001
Systolic Pressure	123.2 ± 11.9	120.6 ± 11.8	0.13	129.9 ± 13.1	125.1 ± 13.9	0.13	0.73
Diastolic Pressure	72.4 ± 8.6	69.6 ± 6.3	0.02	78.5 ± 9.7	78.5 ± 11.6	0.99	0.01
C-Reactive Protein	4.6 ± 4.7	5.0 ± 5.7	0.35	5.3 ± 5.9	4.8 ± 4.1	0.21	0.14
Metabolic Syndrome Score	2.0 ± 0.9	1.7 ± 0.7	0.02	2.5 ± 1.1	2.3 ± 0.9	0.36	0.60

	Weight loss ≥5%			Weight loss ≥5%			p for Difference in mean change between groups
	Baseline	Final	*p*-value	Baseline	Final	*p*-value	
Low KCAL Diet	*N* = 24			*N* = 25			
Glucose	137.9 ± 43.5	105.8 ± 26.1	0.004	144.6 ± 34.3	113.6 ± 23.5	<0.001	0.40
Insulin	26.1 ± 10.9	37.3 ± 29.4	0.08	30.7 ± 14.3	37.3 ± 28.3	0.40	0.88
HOMA-IR	7.9 ± 3.6	7.6 ± 5.3	0.87	10.1 ± 4.4	11.5 ± 9.1	0.56	0.21
LDL-Cholesterol	108.4 ± 30.2	109.5 ± 28.3	0.89	96.4 ± 28.7	96.8 ± 28.0	0.93	0.64
HDL-Cholesterol	40.8 ± 9.8	43.1 ± 10.9	0.04	43.6 ± 10.9	41.0 ± 9.0	0.04	0.86
Triglycerides	157.3 ± 63.1	136.5 ± 49.8	0.09	141.1 ± 63.1	127.2 ± 57.2	0.20	0.67
TG/HDL	3.9 ± 2.0	3.5 ± 1.5	0.27	3.5 ± 1.9	3.3 ± 1.8	0.61	0.96
Systolic Pressure	120.8 ± 14.3	112.2 ± 10.3	<0.001	125.0 ± 14.5	121.3 ± 14.5	0.21	0.03
Diastolic Pressure	67.5 ± 9.0	63.3 ± 6.2	0.01	70.7 ± 9.6	69.1 ± 9.8	0.43	0.04
C Reactive Protein	4.7 ± 3.6	3.8 ± 3.1	0.04	7.6 ± 8.3	8.1 ± 10.3	0.81	0.20
Metabolic Syndrome Score	3.2 ± 0.9	2.5 ± 0.7	0.006	3.0 ± 1.0	2.7 ± 1.0	0.21	0.35

## 4. Discussion

Although some public and scientific debate continues, this study and the cumulative evidence does not support recommending a particular diet type for weight loss – many different types of diets yield significant and clinically meaningful weight loss. Indeed, a meta-analysis of controlled feeding studies evaluating the impact of diet composition (isocaloric low fat *vs* low carbohydrate diets) on daily energy expenditure showed such small differences by diet type that they were physiologically meaningless (26 kcal/day) (24). The present data support the concept that a person can choose a weight loss diet based on individual preference for diet type – indicating that there is no one specific optimal or ideal diet for weight loss. However, the present study provides unique information regarding the demographic, body composition, cardiometabolic, and dietary factors that are associated with the greatest weight loss success within three commonly employed diet types.

The demographic factors of age, self-reported race, and education status were significant predictors of the amount of weight loss and weight loss success (defined as ≥5% of baseline weight). Participant age was associated with weight loss success in both the LOW CHO and LOW KCAL groups. Interestingly, a study in adults age ≥ 25 years showed the amount of weight loss increased with age and older participants were more successful in maintaining weight loss after 3 years (25). Qualitative investigation has provided the insight that engaging in diet change is more likely to be interrupted by lifestyle behaviors, perceived stress, finances, and time challenges in younger adults (26). In contrast, social support, diminishing responsibilities, and greater available time facilitate successful weight loss in older adults (27). Further, doubly labeled water studies show that the age-related decline in resting energy expenditure, which would inhibit weight loss and increase risk for weight gain, does not begin until after age 60 (28). The association of self-reported race with weight loss in the LOW CHO diet is consistent with previous research showing that participants who identified as African American, especially females, achieve less weight loss than white participants - despite similar reported dietary intakes (29–31). It is important to recognize that the racial/ethnic category of “African-American” represents a diverse group of people. Differences in response to diet may be influenced by genetics, cultural influences, family support, finances, living environment, and retention rates (29). It is also remains plausible that the difference in weight loss response by racial/ethnic category is a function of metabolic adaptation related resistance to weight change or dietary compliance (32, 33).

As a biological variable, it has been suggested that sex differences may partly explain the variability in weight loss. In the present study, we did not detect differences by sex with regard to adherence to the diet types and sex was not a significant predictor for the amount of weight loss, for having weight loss success, or for diet intervention completion. These findings contrast with a secondary analysis of the DietFits trial which showed that males were more adherent and lost more weight on a low carbohydrate diet than females (34). It is plausible that differences between males and females in body mass and composition, energy expenditure, as well as dietary preferences, were contributing factors. A systematic review of the published evidence identified only 4 studies designed to directly compare diet-induced weight loss between males and females (35). The difference in amount of weight loss was no longer significant when adjusted for baseline weight in two of the 4 studies, and none of the studies showed significant differences when percent weight change was the outcome.

The influence of educational status on health outcomes such as comorbidities and life expectancy has been well-established (36, 37). However, data from 196,000 participants enrolled in work-based online weight loss programs showed that education level was not a predictor of percent weight loss (38). Yet, in the present findings, education status was a more robust predictor than age or self-reported race. The link between education status and weight loss success may be a function of available income and/or having resources to support diet change. Further, the relationship between education and cognitive function may be a factor influencing diet intervention adherence and the amount of weight loss (39). Nevertheless, lower educational status appears to be related to greater risk for weight gain and obesity (40, 41).

The baseline percentage of body fat, indicating whole body adiposity, was associated with weight loss in the LOW CHO and LOW FAT groups. Population-based evidence shows that the probability of achieving weight loss success, as defined in the present study, increases with higher BMI (42). Interestingly, baseline serum leptin level, which is dependent on total body fat (43, 44), was significantly associated with amount of weight loss in the LOW FAT and LOW KCAL diet groups. It is understood that obesity, and thus, high levels of circulating leptin, are associated with being in a state of leptin resistance that impairs sensitivity to the action of leptin on reducing food intake and increasing energy expenditure (45, 46). Hence, leptin resistance has a role in weight gain and maintaining a higher body weight, and circulating levels of leptin decrease with weight loss (47). Thus, in the present study, individuals with higher BMI but lower leptin levels had greater weight loss. Other forces driving leptin resistance include inflammation and high levels of circulating lipids, and reduced circulating leptin is a predictor of changes in oxidized LDL levels (48). In the present data, both baseline LDL-cholesterol levels and blood pressures associated with the amount of weight loss in the LOW FAT and LOW KCAL groups. It is expected that weight loss would reduce LDL-cholesterol, specifically the prevalence of small dense LDL particles (49), and improve blood pressure. However, whether baseline levels of LDL-cholesterol or baseline blood pressure would influence achieving weight loss has not been investigated. It is likely that the type of diet interventions provided attracted participants who are motivated by concern for their cardiometabolic risk and seek the benefits of weight loss on commonly measured clinical risk factors (50).

The most robust predictor of successful weight loss was completion status, accounting for 20–30% of the variance in weight loss. Like other non-surgical interventions for weight management, the attrition rate averaged 20–30% for the three diet types (51, 52). An ongoing challenge for weight management interventions is how to improve participant retention. Indeed, the World Health Organization has deemed adherence to treatments for chronic disease states a critical problem (53). We found that the amount of weight loss being achieved was the most robust predictor of study completion. Consistent with this finding, meta-analysis of 10 studies showed that dissatisfaction with weight loss results was associated with lower adherence (54). As with the present findings, older age was also associated with higher adherence. Given the widespread prevalence of obesity and increasing burden of cardiometabolic disease in younger adults, it remains crucial to identify efficacious treatment approaches for adults in their 20s, 30s and 40s (55).

Notably, participants enrolled in all three diet types demonstrated valuable improvements in several cardiometabolic risk factors, especially those who achieved weight loss success (≥ 5% baseline weight). A modest 5% reduction in weight has been accepted as clinically meaningful as it is associated with improved percentage body fat, reduced intra-abdominal and intra-hepatic fat, reduced circulating levels of glucose, insulin, and triglycerides, as well as reduced HbA1C and blood pressures (56). Participants who achieved weight loss success in the three diet types experienced significant improvements in 4–5 key cardiometabolic risk factors. Weight loss success in all three diet types was associated with reduced blood pressure, although the improvement in the LOW FAT group was detected only for diastolic blood pressure. In addition to improved blood pressure, participants in the LOW FAT group experienced improved LDL-cholesterol, triglycerides, and TG/HDL ratio. Participants in the LOW CHO group experienced improvements in insulin levels, HOMA-IR score, and LDL-cholesterol. Participants in the LOW KCAL group experienced the greatest improvements in circulating glucose, HDL-cholesterol, and C-reactive protein.

This study has several limitations and strengths to be noted. First, the study was not a randomized controlled trial which would limit bias and allow for determination of cause and effect. Instead, participants chose the diet type they preferred, which mimics real-world conditions where almost half of all adults age 20 and over are attempting to lose weight by modifying their dietary intakes and/or physical activity (57). Second, the dataset did not include all the factors that may contribute to predicting the amount of weight loss or weight loss success, including genetic differences and some cognitive, behavioral, and environmental components of eating. Nevertheless, the inclusion of a wide variety of biological, physiological, and psychological factors into predictive modeling enabled accounting for a significant portion of the variance in both the amount of weight loss and achieving weight loss success. Third, we recognize the limitation of under-reporting in diet assessment. To reduce this bias, we train study subjects on portion size estimation using food models and measuring utensils, and we incorporate multi-pass methodology along with NDSR software generated prompts. Fourth, the diet intervention period was 4–6 months which limits extrapolating the findings to long-term intervention or weight loss maintenance. Finally, the addition of exercise combined with diet intervention may provide the stimulus for greater response, and thus, warrants future investigation.

## 5. Conclusion

The findings from this study support the concept that completion of an intervention to improve weight and health is the most important factor for a successful outcome. The data show that successful weight loss can be achieved through various dietary strategies. However, reducing weight to positively impact specific cardiometabolic risk factors differs by type of diet intervention. These findings indicate that a low carbohydrate diet may be most optimal for individuals at risk for prediabetes or type 2 diabetes. A low-fat diet may be most beneficial for reducing atherogenic dyslipidemia. A calorically restricted diet may improve either condition. Aligning a diet intervention type with an individual’s personal risk factors is likely the most efficacious approach for improving cardiometabolic health.

## Data availability statement

The raw data supporting the conclusions of this article could be made available by the authors, without undue reservation.

## Ethics statement

The studies were approved by the Vanderbilt University Medical Center Institutional Review 66 Board and all participants signed written informed consent. The patients/participants provided written informed consent prior to their study participation.

## Author contributions

HS and EG: study concept, study design, and funding. HS: data acquisition and database entry. JL: statistical analysis. JL, EG, and HS: tables, figures and manuscript development. JL, MK, EG, and HS: manuscript revisions and final draft. All authors contributed to the article and approved the submitted version.

## Funding

Article processing charges were funded by Louisiana State University Agricultural Center.

## Conflict of interest

The authors declare that the research was conducted in the absence of any commercial or financial relationships that could be construed as a potential conflict of interest.

The handling editor GB declared a shared affiliation with the author HS at the time of review.

## Publisher’s note

All claims expressed in this article are solely those of the authors and do not necessarily represent those of their affiliated organizations, or those of the publisher, the editors and the reviewers. Any product that may be evaluated in this article, or claim that may be made by its manufacturer, is not guaranteed or endorsed by the publisher.
